# Self-Monitoring Artificial Red Cells with Sufficient Oxygen Supply for Enhanced Photodynamic Therapy

**DOI:** 10.1038/srep23393

**Published:** 2016-03-18

**Authors:** Zhenyu Luo, Mingbin Zheng, Pengfei Zhao, Ze Chen, Fungming Siu, Ping Gong, Guanhui Gao, Zonghai Sheng, Cuifang Zheng, Yifan Ma, Lintao Cai

**Affiliations:** 1 Guangdong Key Laboratory of Nanomedicine, CAS Key Lab for Health Informatics, Institute of Biomedicine and Biotechnology, Shenzhen Institutes of Advanced Technology (SIAT), Chinese Academy of Sciences, Shenzhen 518055, PR China; 2 Department of Chemistry, Guangdong Medical University, Dongguan 523808, PR China; 3 Center for High Performance Computing, Institute of Advanced Computing and Digital Engineering, Shenzhen Institutes of Advanced Technology, Chinese Academy of Sciences, Shenzhen 518055, PR China; 4 University of Chinese Academy of Sciences, Beijing 100049, PR China

## Abstract

Photodynamic therapy has been increasingly applied in clinical cancer treatments. However, native hypoxic tumoural microenvironment and lacking oxygen supply are the major barriers hindering photodynamic reactions. To solve this problem, we have developed biomimetic artificial red cells by loading complexes of oxygen-carrier (hemoglobin) and photosensitizer (indocyanine green) for boosted photodynamic strategy. Such nanosystem provides a coupling structure with stable self-oxygen supply and acting as an ideal fluorescent/photoacoustic imaging probe, dynamically monitoring the nanoparticle biodistribution and the treatment of PDT. Upon exposure to near-infrared laser, the remote-triggered photosensitizer generates massive cytotoxic reactive oxygen species (ROS) with sufficient oxygen supply. Importantly, hemoglobin is simultaneously oxidized into the more active and resident ferryl-hemoglobin leading to persistent cytotoxicity. ROS and ferryl-hemoglobin synergistically trigger the oxidative damage of xenograft tumour resulting in complete suppression. The artificial red cells with self-monitoring and boosted photodynamic efficacy could serve as a versatile theranostic platform.

In past decades, photodynamic therapy (PDT) has emerged as a promising interventional treatment for cancer therapy. The goal of a successful PDT is to evoke a potent and sustained photo-chemical reactions among light, photosensitizer and tissue oxygen^1^. Upon light irradiation, the photosensitizer converts nontoxic oxygen into cytotoxic reactive oxygen species (ROS) to destroy tumour cells and vasculature^2^. Unfortunately, the clinical application of PDT remains limitations in some extend due to potential adverse effects and poor tumour accumulation of photosensitizers. Nanoparticle-based delivery system has been developed as an effective strategy to increase the accumulation efficiency and selectivity of photosensitizer in tumour^3^,^4^. During photodynamic reaction, the sufficient oxygen source is highly desirable for a successful PDT. Paradoxically, hypoxia is a hallmark of most solid tumours because of the poor vascular architecture from preexisting venules^5^. Furthermore, the consumption of oxygen during PDT aggravates tumour hypoxia^6^,^7^, which potentially dampens the therapeutic effect of oxygen-dependent PDT. To overcome this hypoxia limitation, various strategies have been explored, including combination with other photodynamic mechanisms, or catalyzing intracellular H_2_O_2_ to O_2_ 8,9.

Hemoglobin (Hb) is an iron-rich protein in red blood cells that delivers oxygen to tissues, which could reversibly bind 4 oxygen molecules to form HbO_2_ 10. But cell-free Hb is not suitable for an ideal oxygen donor because it produces severe problems, including short circulation time, potential side effect, and poor stability^11^. As a potential new class of oxygen carrier, Hb-based oxygen carrier *via* encapsulation with biodegradable materials could successfully conquer the defects of cell-free Hb and obtain the similar oxygen-carrying capability as natural red cells^12^,^13^. Better than red cells, nano-dimensional oxygen carriers can perfuse tumour tissues within the narrow vascular structure, which can supply more oxygen in hypoxic tumour^14^,^15^. It is worthy of note that iron-rich Hb can be easily oxidized into highly oxidative intermediates so called ferryl-Hb species, resulting in cell injuries by oxidative heme iron^16^,^17^,^18^,^19^.

Herein, we developed a biomimetic lipid-polymer nanoparticle, loading complexes of photosensitizer (indocyanine green, ICG) and oxygen-carrier (Hb), acting as nano-sized artificial red cells to incorporate oxygen supply and monitor the treatment of photodynamic process. These ICG-loaded artificial red cells (I-ARCs), consisted of a lipid layer of lecithin and 1,2-distearoyl-sn-glycero-3-phosphoethanolamine-N-maleimide (polyethylene glycol 2000) (DSPE-PEG) and a core of poly(lactic-*co*-glycolic acid) (PLGA) encapsulating Hb/ICG complex (Fig. 1a). The lipid layer was designed to mimic the cell membrane, and PLGA-encapsulated Hb could reversibly bind 4 oxygen molecules to form HbO_2_, with oxygen carrying function similar to red cells. Upon near-infrared (NIR) laser irradiation, ICG could effectively convert nontoxic oxygen to cytotoxic ROS with efficient oxygen supplement. In the meantime, ROS could oxidize ambient ferrous-Hb to cytotoxic ferryl-Hb species (Fig. 1b). The outbreak of ROS and far more lasting damage of ferryl-Hb species eventually triggered to boosted destruction to tumours (Fig. 1c). Moreover, the fluorescence (FL) of ICG and photoacoustic (PA) response of ICG, HbO_2_ and Hb would dynamically self-monitor the biodistribution and metabolism of components in I-ARCs during PDT.

## Results

### Structure-interaction of Hb/ICG complexes and characterization of I-ARCs

When ICG was introduced to clinics, it was reported that more than 98% of ICG bound to proteins (lipoproteins, albumin, *etc.*) in blood, leading to rapid aggregation and clearance from the body^20^,^21^. We investigated binding properties and molecular interactions between ICG and Hb by computational chemistry using the Internal Coordinate Mechanics (ICM) program (Molsoft). The result revealed that, Hb was made of 4 polypeptide subunits, and ICG occupied the interface close to subunit A and B of the Hb, and the distances from ICG to the four hemes were 17.3, 18.4, 25.4 and 21.3 Å respectively (Fig. 2a). Each ICG was surrounded by 10 amino-acid residues with extensive electrostatic and hydrophobic interactions, which formed shell interaction with ICG (Figs 2b, S1a). Consistent with previous report (in electrophoresis, ICG stayed in bands of plasma protein), the result of native-PAGE electrophoresis and FL detection revealed the co-localization of ICG and Hb, which evidenced the formation of ICG/Hb complexes (Fig. S1b)^20^. The Hb/ICG complexes significantly enhanced the encapsulation efficiency to over 95% of both Hb and ICG, compared to Hb (86%) or ICG (76%) alone. Moreover, the proximal distance between ICG and hemes facilitated not only rapid oxygen transport to ICG in photodynamic process, but also simultaneously effective Hb oxidization by ROS, which provided a fundamental basis for the enhanced PDT.

**Figure 1:**
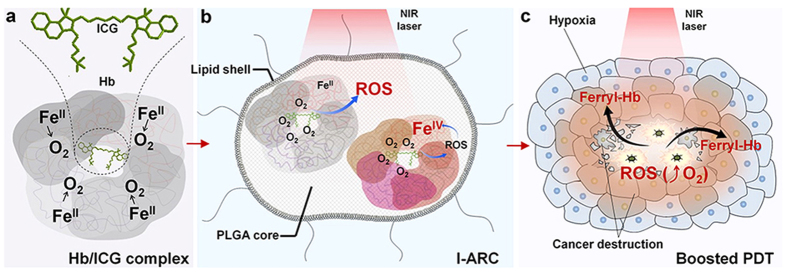
Schematic illustration of cancer boosted photodynamic therapy (PDT) based on ICG loaded artificial red cells (I-ARCs). (**a**) Hb/ICG complex: Hemoglobin (Hb) and indocyanine green (ICG) bound together through electrostatic and hydrophobic interactions for Hb/ICG complex. One Hb molecule reversibly bound 4 oxygen molecules, supplying abundant oxygen for PDT treatment. (**b**) ICG-loaded artificial red cell (I-ARC): The PLGA core in ICG loaded I-ARCs encapsulated photosensitizer (ICG) and oxygen supplier (Hb), and the lecithin and DSPE-PEG covered the core to form a lipid membrane for bioinspired I-ARCs. (**c**) Boosted PDT: Oxygen supply promoted reactive oxygen species (ROS) generation in NIR laser triggered PDT, and ROS spontaneously converted ferrous-Hb (Fe^II^) into cytotoxic ferryl-Hb (Fe^IV^), promoting the lifetime of oxidative destruction for boosted PDT. The synergistic effects of ROS and ferryl-Hb caused irreversible oxidative damage of cancer by breaking its hypoxia microenvironment with I-ARCs.

**Figure 2:**
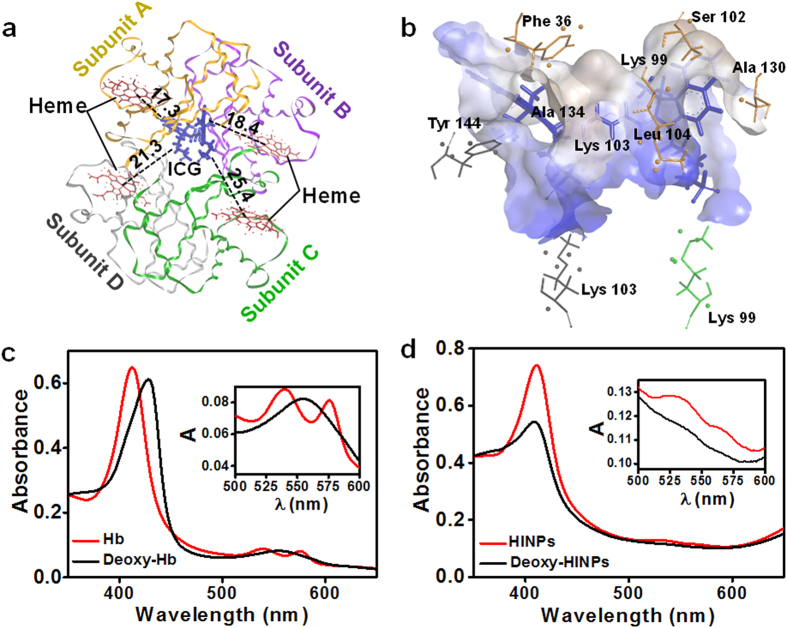
The molecular interaction model of Hb/ICG complex, and characterization of the I-ARCs. (**a**) Structural diagram of ICG and Hb complex. Hb consisted of four polypeptide subunits, and ICG molecule bound at the site close to subunit A (yellow) and subunit B (purple) of Hb. ICG was surrounded by 4 hemes, and the oxygens carried on hemes were easily transferred to boosted PDT. (**b**) The hydrophobic receptor surface was shown as semitransparent surface coloured according to binding properties: blue for hydrophilic and brown for hydrophobic. (**c**) UV-Vis absorption spectra of deoxy-/oxy- Hb. Inset image in detail showed the characteristic absorption of deoxy-/oxy- Hb. (**d**) UV-Vis absorption spectra of I-NARCs and deoxygenated I-NARCs. Inset image in detail showed the absorption of deoxy-/oxy- I-NARCs, in which the characteristic absorption changed similarly to that of deoxy-/oxy- Hb. (**e**) Acidic stability of Hb in I-ARCs. Precipitation occurs in Free Hb solution at pH 6.7, significantly reduced oxygen loading function, while nano-functionalized Hb solution (I-ARCs) kept clear and transparent, proving a wonderful stability under acidic condition. (**f**) Thermal stability of Hb in I-ARCs, ARCs, or free Hb.

Transmission electron microscopy (TEM) and dynamic light scattering (DLS) demonstrated that I-ARCs had a typical spherical shape with good monodispersity and a diameter around 70 nm (Fig. S2). Furthermore, I-ARCs also exhibited superior size durability and highly enhanced fluorescent stability of ICG (Fig. S3). Abundant oxygen in tumour tissues is required for effective ROS generation and successful PDT. Before the oxygen loading tests of I-ARCs, pure oxygen was poured into nanoparticle solutions for oxygenation. The reversible combination of Hb and O_2_ in I-ARCs was confirmed that deoxy-Hb had the characteristic single peak and oxy-Hb showed double peaks (Fig. 1c,d). The oxygen loading function of I-ARCs was visually assessed by measuring the PA signals of HbO_2_ and Hb. After oxygenation, I-ARCs had relatively high oxygen saturation (Fig. S4a). Moreover, The Hb in I-ARC showed up the resistance against high temperature (60 °C) and pH 6.7 environment (extracellular pH in tumour is mild acidic (6.5–7.2)), where cell-free Hb might denaturate or precipitate, and consequently lose its oxygen carrying function (Fig. 2e,f)^22^. In a hypoxic solution, I-ARCs demonstrated a steady oxygen release with the semi-dissociation time at 46 min, which was significantly longer than that of free Hb (5.2 min), oxygen commendably sustained within I-ARCs (Fig. S4b).

### I-ARCs as an efficient promoter for boosted PDT *in vitro*

**Figure 3:**
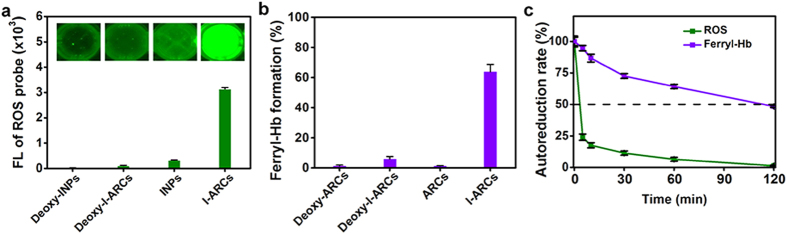
ROS generation and ROS-induced ferryl-Hb formation in I-ARCs. (**a**) Evaluation of ROS yield. The FL signal of I-ARCs is 9.5 times stronger than that of INPs. (**b**) Quantification of ferryl-Hb in INPs and I-ARCs. In I-ARCs, 63.8% ferrous-Hb (Fe^II^) was converted into ferryl-Hb (Fe^IV^), which is 10.7 times higher than that of deoxygenated I-ARCs. (**c**) Comparison of autoreduction time of ROS and ferryl-Hb produced in I-ARCs after NIR laser irradiation. The half-autoreduction time of Ferryl-Hb is 35-fold longer than that of ROS.

The ROS and ferryl-Hb generated in photodynamic process were quantified. With laser irradiation, oxygen-loaded I-ARCs released significantly higher ROS than deoxy-I-ARCs and deoxy-ICG nanoparticles (INPs), where the ROS yield of I-ARCs was 9.5 times higher than that of INPs (Fig. 3a). Without oxygen, laser-triggered ROS production was barely detectable, which illustrated that abundant ROS generation in PDT relied on sufficient oxygen supplying. Ferryl-Hb as the product of Hb oxidization was evaluated in different nanoparticles (Fig. 3b). Without photosensitizer, artificial red cells (ARCs) hardly formed ferryl-Hb under laser irradiation. Without oxygen, the generation rate of ferryl-Hb in I-ARCs was less than 6%, whereas the ferryl-Hb in I-ARCs dramatically increased to 63.8% upon laser irradiation. It indicated that, the bounteous ROS production was essential for ferryl-Hb conversion. As reported, the half-life of ferryl-Hb was prolonged by several orders of magnitude, compared to ROS^23^,^24^,^25^. The level of ROS in I-ARCs quickly declined in solution within 5 min after laser irradiation, confirming its short life span, but the level of ferryl-Hb in I-ARCs remained 94.8% under the same conditions (Fig. 3c). Consistent with the previous reports^23^,^24^,^25^, the half autoreduction time of ferryl-Hb was 35-fold longer than that of ROS, and the cytotoxicity of ROS could be greatly promoted by oxidizing Hb to cytotoxic ferryl-Hb in I-ARCs mediated PDT. Due to the prolonged lifetime, ferryl-Hb could diffuse a much longer distance in cells than ROS, thus creating a far more persistent oxidative damage, subsequently led to cell death^26^.

**Figure 4:**
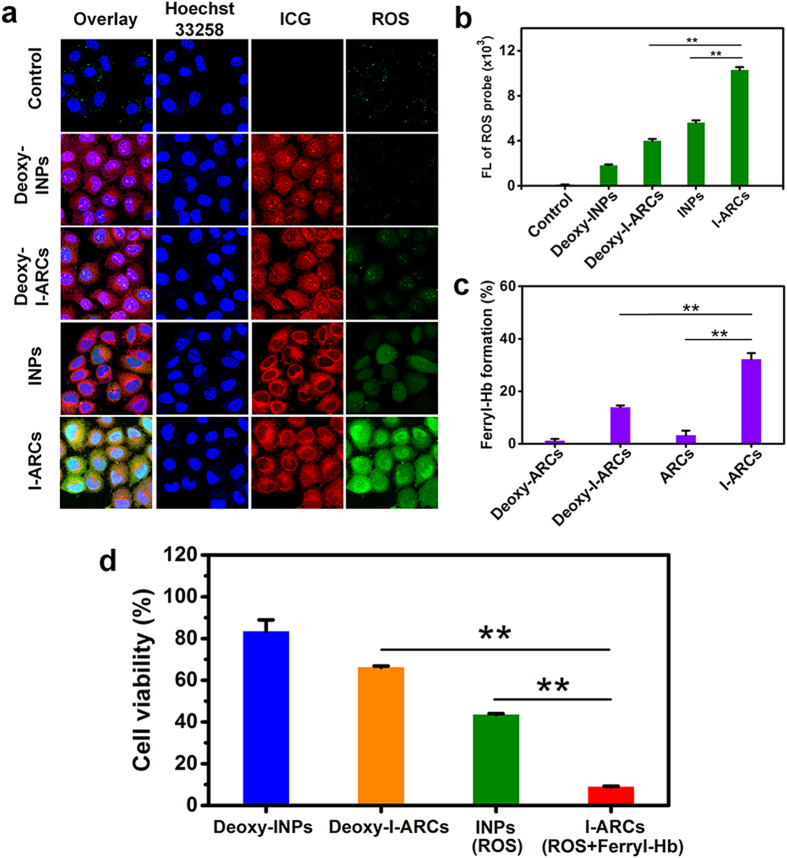
The boosted PDT effect of I-ARCs for cancer cell *in vitro*. Cellular ROS detection after PDT measured by (**a**) confocal microscopy and (**b**) flow cytometry. Due to the oxygen-loaded Hb presence, I-NARCs generated abundant ROS that could produce oxidative damage in the cells. *P < 0.05, **P < 0.01. (**c**) Quantitative test of ferryl-Hb in the cell homogenate. *P < 0.05, **P < 0.01. (**d**) The cell viability was detected at 24 h cell incubation after laser treatments.

PDT-triggered intracellular ROS generation and intracellular ICG were detected using confocal microscopy and flow cytometry. After laser irradiation, deoxy-INPs, deoxy-I-ARCs and oxygen-filled INPs produced a small quantity of ROS, while the mean FL intensity of ROS in I-ARCs group was 1.8 ~ 5 times stronger (Figs 4a,b, S5). It was notable that INPs produced ROS less effectively than I-ARCs did, indicating that the direct oxygen filling was not effective to improve the photodynamic efficiency. The amount of ferryl-Hb was also assessed in MCF-7 cells after PDT. Hb nanoparticles (ARCs) was hard to convert ferrous-Hb into ferryl-Hb in the absence of ICG. In contrast, the treatment of I-ARCs showed about that 32.2% of Hb was oxidized into ferryl-Hb, which was 2.3-fold higher than deoxy-I-ARCs did (Fig. 4c). These results were consistent with the observation in solution, and further confirmed Hb in I-ARCs as an effective oxygen donor and specific ferry-Hbs to boost the photodynamic reactions.

The I-ARCs mediated PDT was further visually evaluated at the different nanoparticles-mediated PDT using the loading amount of 100 μg mL^−1^ ICG with or without 804 μg mL^−1^ Hb under laser irradiation. MCF-7 cells were stained with calcein-AM or propidium iodide (PI) to identify live or dead/late apoptotic cells. Laser alone or deoxy-I-ARCs plus laser caused a few cell deaths, while oxygen-loaded I-ARCs plus laser caused obvious cell death as demonstrated by observing PI stained cells (Fig. S6). Cell viability was further evaluated, the viability of MCF-7 was not significantly affected in the treatment groups of deoxy-INPs (83.5%) and deoxy-I-ARCs (66.2%), confirming the indispensible role of oxygen in PDT (Fig. 4d). Without oxygen-carrying Hb, INPs could only slightly decrease the viability to 43.5%. Remarkably, cell viability decreased to 8.9% in I-ARCs group, for the enhanced both of ROS production and ferryl-Hb generation, which effectively and synergetically promoted the cytotoxicity.

### Dynamic self-monitoring of biodistribution and PDT process of I-ARCs *in vivo*

**Figure 5:**
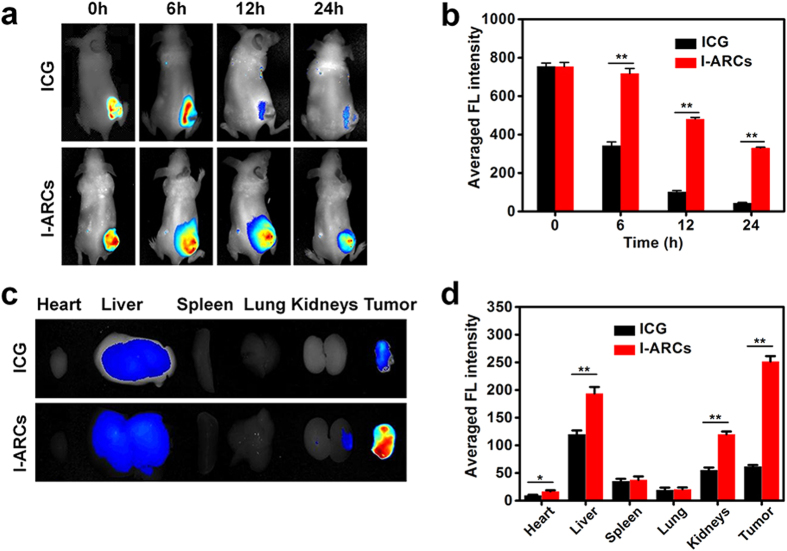
The biodistribution and metabolism of I-ARCs *in vivo*. (**a**) Time-lapse NIR FL images of nude mice after intratumoural administration of free ICG or I-NARCs. (**b**) Quantified NIR FL intensities around the tumour at indicated time points. *P < 0.05, **P < 0.01. (**c**) NIR FL images of major organs and tumours after injection of free ICG or I-NARCs at 24 h. (**d**) Semiquantitative biodistribution of free ICG or I-NARCs in nude mice determined by the averaged FL intensity of organs and tumour. *P < 0.05, **P < 0.01.

The biodistribution of I-ARC was examined by determining the ICG fluorescence with the *in vivo* imaging system. The FL intensity of ICG in the tumour of I-ARCs group declined much less than that of free ICG, indicating superior tumour retention of ICG by nanoparticles (Fig. 5a). At 6 h, the FL intensity of ICG in I-ARCs treated mice was 95% of initial intensity, which was 2.1-fold higher than that of free ICG (Fig. 5b). The metabolism of ICG in major organs were analyzed at 24 h. The result showed that both free ICG and I-ARCs entered blood circulation, and mostly accumulated in the tumour and liver, little in kidneys (Fig. 5c). Mice injected with I-ARCs showed higher ICG retention in tumour, which was 3.5 times as compared to free ICG group (Fig. 5d). I-ARCs prevented the metabolism of ICG photosensitizer *in vivo*, indicating enhanced retention of ICG for at least 6 h, which was attributed by nanoparticle encapsulation. These could be also relative to the strong interaction between Hb and ICG and the passive targeting with enhanced tumour retention of I-ARCs.

**Figure 6:**
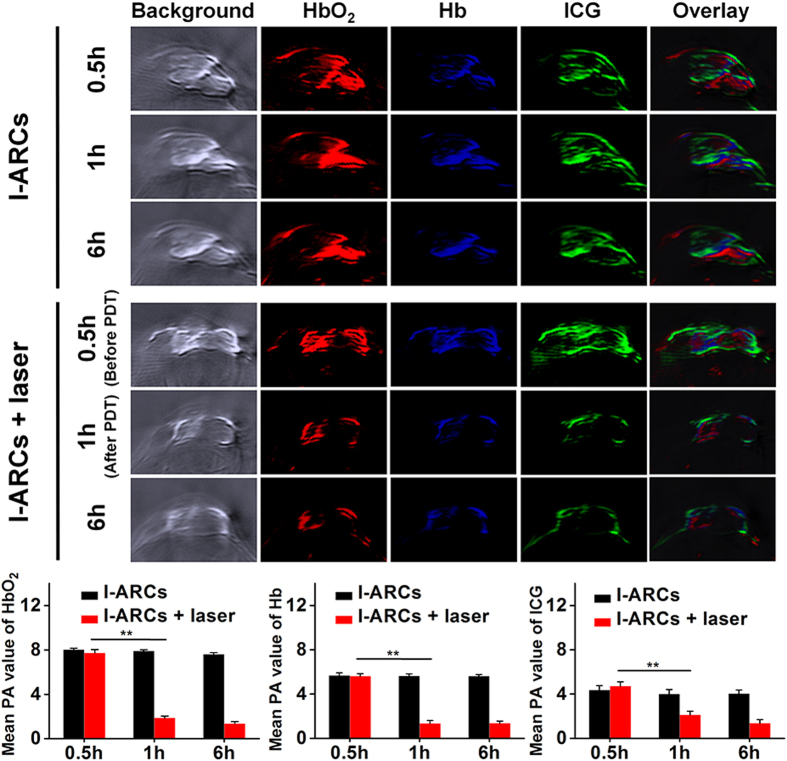
*In vivo* real-time self-monitoring oxygen/photosensitizer consumption in tumour by intrinsic PA response of HbO_2_, Hb and ICG in I-NARCs using MSOT system. *P < 0.05, **P < 0.01.

Moreover, the dynamical PDT process can be self-monitored on ICG/oxygen levels in the tumour region via the PA response of ICG, HbO_2_ and Hb in I-ARCs. Multiple spectral optoacoustic tomography (MSOT) was used to semiquantitatively measure the amount of ICG, HbO_2_ and Hb in tumours for 6 h after intratumoural injection. At 0.5 h after injection, I-ARCs elicited a strong PA response in tumours. The PA intensity of HbO_2_ in I-ARCs groups were over 15 times higher than that in PBS group, indicating significantly elevated oxygen level in tumours (Figs 6, S7). Without NIR laser irradiation, the PA intensity of HbO_2_, Hb and ICG in I-ARCs did not significantly change within 6 h, indicating that I-ARCs effectively held both photosensitizer and oxygen in tumours, which was critical for achieving highly efficient PDT (Fig. 6). After 30 min NIR-irradiation (1 h post injection), the PA values of HbO_2_, Hb and ICG significantly decreased to 24.2%, 24.0% and 45.3%, respectively (Fig. 6). The declined PA signal of HbO_2_ in tumours was attributed to the oxygen consumption by NIR triggered photodynamic process. In the meantime, the PA signal of Hb was also declined, which might be related to the oxidization from ferrous-Hb to ferryl-Hb as previously observed in solution and *in vitro* (Fig. 6). The PA signal of ICG also dramatically decreased upon NIR laser irradiation, which was due to the consumption and bleach of photosensitizer (Fig. 6). The results indicated that the I-ARCs effectively retained photosensitizer and greatly increased oxygen level in tumour region. In summary, the FL and PA dual-modal imaging of I-ARCs not only dynamically self-monitored the O_2_/ICG consumption during the PDT process, but also predicted the nanoparticle biodistribution and the efficiency of PDT *in vivo*, thereby facilitating the image-tracking and monitoring cancer phototherapy.

### Anti-tumour effect and biosafety of I-ARCs *in vivo*

To evaluate the anti-cancer effect of PDT, mice bearing MCF-7 tumours were intratumourally injected with I-ARCs, followed by NIR laser irradiation under low power (0.1 W cm^−2^) for 30 min, in order to maintain the temperature less than 41 °C to avoid exceeding the photothermal damage threshold of cancer cell^27^. As a biosafe photosensitizer, ICG has been clinically used for PDT to treat skin diseases and bacterial infections under low power NIR laser^28^,^29^. In fact, intratumoural injection was widely applicable as interventional treatment in clinical tumour treatment, but essential for bypassing much of the preexisting barriers associated with systemic intravenous injection. It has been reported that Gendicine^®^ (gene therapy medicine), HF10 (oncolytic virus), *etc*. by intratumoural injection can successfully suppress breast, head and neck, or pancreatic tumour with less side-effects in clinical applications^30^,^31^.

**Figure 7:**
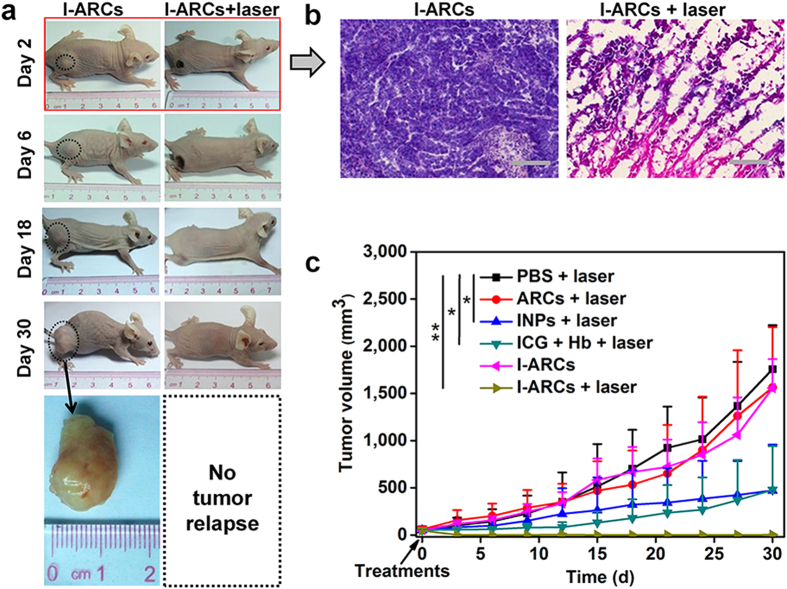
The anti-tumour evaluation of I-ARC based PDT. (**a**) Representative photos of mice bearing MCF-7 tumours on 30 d after treatments. The tumour regions were marked with dashed circles. Rapid growth of tumour occurred in the group without NIR laser irradiation, the laser treated group had a complete remission of MCF-7 tumours, leaving the original tumour site with black scars which fell off about 18 d later. (**b**) H & E stained histological sections of tumour revealed antitumour activity of boosted PDT (scale bar, 100 μm). (**c**) MCF-7 tumour growth curves of different groups after treatments. *P < 0.05, **P < 0.01. All injectants were oxygenated before experiments.

Eventually, antitumour effect of I-ARCs mediated PDT was monitored after PDT. Tumours treated with PBS + laser grew rapidly, suggesting that the MCF-7 tumour growth was not affected by NIR laser irradiation (Figs 6c, S8). As expected, ARCs + laser or I-ARCs without laser did not affect tumour growth as well because of lacking photosensitizer or laser (Figs 7a,c, S8). Two days after laser irradiation, the mice treated with I-ARCs showed severe tumour destruction with black scar at the tumour region (Fig. 7a). The hematoxylin and eosin (H&E) staining of tumour sections in I-ARCs + laser group demonstrated severe tumour destruction accompanied with necrotic cellular debris and dramatically reduced tumour cells (Figs 7b, S9). Remarkably, a single injection of I-ARCs plus NIR laser irradiation exceptionally led to a complete remission of MCF-7 tumours, leaving the original tumour site with black scars which fell off about 2 weeks later (Fig. 7a,c). No tumour recurrence was observed in this group over a course of 30 d (Fig. 7a). Therefore single dose I-ARCs-mediated PDT effectively suppressed tumour growth and prevented tumour recurrence. Due to the synergized effect of *in situ* induced ROS and oxidized ferryl-Hb in tumour, the anti-cancer effect of PDT was strongly enhanced by I-ARCs.

Comparing with I-ARCs + laser, the treatment of INPs + laser inhibited tumour growth less effectively, which should be due to the absence of oxygen donor (Hb) and hypoxic condition in tumour. On the other hand, although free ICG + Hb + laser led to moderate tumour destruction and partially inhibited tumour growth within 6 d post PDT, tumour relapsed afterwards (Fig. S8). The anti-tumour efficacy of free ICG + Hb + laser was inefficient, which could be attributable to the poor tumour retention and instability of free ICG and Hb, thereby diminishing the efficiency of PDT in tumours. However, without NIR laser irradiation, I-ARCs did not affect tumour growth. The change of body weight of mice were recorded during the treatments, these groups were not significantly different from the control group, suggesting that these treatments were well tolerated. We further investigated the potential toxicity of the I-ARCs in the kidneys and liver which had significant accumulation of nanoparticles. The H&E stained organ sections with no apparent histological changes confirmed the great biocompatibility and biosafety of I-ARCs (Fig. S10).

## Discussion

In PDT, particular attention has been focused on oxygen supply, which is a crucial fact to determine the final effect of PDT. However, native hypoxic tumoural microenvironment is a major barrier for potent PDT. Therefore, seeking safe and effective strategies of oxygen carrying to hypoxic tumoural region is desirable. The key idea of our study is the supply of oxygen to the hypoxic tumours through a stable and highly efficient way (ARCs or I-ARCs), so that the PDT action photosensitized by ICG in I-ARCs can be boosted with abundant oxygen source.

The cell-free Hb, a native oxygen carrier, is not a good oxygen carrier for its poor thermo-/pH stability, but is very stable when encapsulated in ARCs. Coincidentally, free ICG itself shows poor photostability in aqueous solution and is rapidly cleared from the body. As ICG and Hb were co-loaded to construct I-ARCs, the formation of ICG/Hb complexes and the encapsulation of nanoparticle system greatly enhanced thermo-/pH stability of Hb and retention time of ICG in cells and tumours. The binding properties between Hb and ICG also make two other advantages in photodynamic process: 1) Hb oxygen donor is tightly bound to photosensitizer so that the oxygen utilization in NIR laser-induced PDT could be greatly enhanced. 2) Owing to the formation of ICG/Hb complexes, the PDT-induced ROS could effectively oxidize Hb to ferryl-Hb with a prolonged lifetime, even though the diffusion range of ROS is rather short. Our results showed that, Hb played a crucial role not only as self-sufficient oxygen donor, but also an amplifier of photodynamic effect when oxidized to ferryl-Hb, because of the much longer lifetime of ferryl-Hb compared to ROS.

I-ARC itself acted as an ideal FL/PA bimodal imaging probe for the treatment of PDT, and thus created a self-monitoring theranostic platform. The long-retained ICG dye due to encapsulation indicated the biodistribution of photosensitizer through FL imaging. Moreover, when I-ARCs were intratumourally injected, PA imaging of tumour mapped hypoxic/hyperoxic regions and photosensitizer (ICG) distribution in the entire tumour, and the PDT process was successfully visualized by monitoring the consumption of ICG and Hb. During photodynamic process, abundant ROS was induced, and Hb itself was *in situ* oxidized into ferryl-Hb which led to prolonged cytotoxicity of tumours. Such synergistic effects of ROS and ferry-Hb dramatically suppressed the tumour growth. The result of PDT-treated mice obtained with I-ARCs was superior to all the other control groups. The I-ARCs are found to be bio-safe/-compatible as well, which increase their potency.

In conclusion, we demonstrated our primary goal: the necessity of sufficient oxygen supply by Hb for efficient PDT against hypoxic tumors, and the self-monitoring PDT through FL/PA dual-modal imaging. Such versatile artificial red cells loading Hb/ICG can offered as a multifunctional theranostic nanoplatform and greatly enhanced PDT for breaking hypoxia in cancer targeted therapy.

## Methods

### Materials

Indocyanine green (ICG), poly(D,L-lactide-co-glycolide) (PLGA, MW, 5000–15000; lactide, glycolide (50:50)), hematoxylin and eosin, Sodium sulfide (Na_2_S), and 2′,7′-dichlorofluorescin diacetate (DCFH-DA) were purchased from Sigma-Aldrich (USA). Bovine hemoglobin (Hb) was purchased from J&K Scientific Ltd. (China). Soybean lecithin, 1,2-distearoyl-sn-glycero-3-phosphoethanolamine-N-maleimide (polyethylene glycol 2000) (DSPE-PEG) were obtained from Avanti (USA). Coomassie Blue, Calcein-AM and Propidium Iodide (PI) Cell Apoptosis Kit were obtained from Invitrogen (USA). Fetal bovine serum, dulbecco’s modified eagle medium (DMEM) and penicillin-streptomycin were purchased from Gibco Life Technologies (USA). 3-(4,5-dimethylthiazol-2-yl)-5-(3-carboxymethoxyphenyl)-2-(4-sulfophenyl)-2H- tetrazolium (MTS) was bought from Promega (USA). Amicon ultra-4 centrifugal filter with a molecular weight cutoff of 100 kDa was bought from Millipore (USA).

### Formulation of ICG nanoparticles (INPs), artificial red cells (ARCs) and ICG-loaded artificial red cells (I-ARCs)

I-ARCs were synthesized using PLGA, soybean lecithin, Hb, ICG, and DSPE-PEG by a previously reported single-step sonication method^3^. PLGA was dissolved in 80% acetonitrile aqueous solution at a concentration of 2 mg mL^−1^. To synthesis I-ARCs, (lecithin)/(DSPE-PEG) (mass ratio was 2:3) with a total mass ratio of 25% to the PLGA polymer, 2000 μg Hb, and 250 μg ICG were added in 3 mL of 4% ethanol aqueous solution, and 1 mL PLGA solution was added dropwise under sonication using an ultrasonics processor (VCX130, USA) at a frequency of 20 k Hz and power of 32 W for 5 min. Finally the I-ARCs were washed three times using a 100 kDa Amicon ultra-4 centrifugal filter. The same procedures were used to prepare the INPs or ARCs in the absence of Hb or ICG. The INPs, ARCs and I-ARCs were deoxygenated by pure argon stream and stored for experiments. For oxygenation or deoxygenation operations, pure oxygen or argon gas ran through the nanoparticle solutions filled in an airtight cell for 1 h, followed by monitoring the absorption spectra to confirm the oxy- or deoxy-state^32^.

### Binding analysis of Hb and ICG

The molecular computation concerning binding between Hb (Protein Data Bank No. 2QSS) and ICG was performed using ICM-Pro program (Molsoft, USA)^33^. Positions of asparagine (Asn) and glutamine (Gln) were optimized to maximize hydrogen bonding. The correct stereochemistry and formal charges were assigned. The ICG was then assigned the Merck Molecular Force Field (MMFF) atom types and charges, and subjected to a global energy optimization using Internal Coordinate Mechanics (ICM) stochastic optimization algorithm. The molecular system was described in terms of internal coordinate variables, using a modified Empirical Conformational Energy Program for Peptides (ECEPP/3) force-field with distance-dependent dielectric constant for the energy calculations as implemented in ICM. For native-PAGE electrophoresis, 20 μL of ICG-Hb mixture (containing 400 μg mL^−1^ ICG and 3200 μg mL^−1^ Hb), ICG (containing 400 μg mL^−1^ ICG) or Hb (containing 3200 μg mL^−1^ Hb) in 0.5 M Tris-HCl buffer (pH 8.8, containing 10% glycerol) was loaded into each well in an XCell SureLock Electrophoresis System (Invitrogen) based on the manufacturer’s instructions. Native-PAGE electrophoresis was realized on 12% polyacrylamide gel at 120 V and 4 °C. Protein staining was accomplished using Coomassie Blue and destained in water overnight before FL imaging. The FL of ICG were detected using IVIS spectrum imaging system (Xenogen, USA).

### Characterization

Size distribution of all nanoparticles was acquired by dynamic light scattering (DLS) using a Malvern Zetasizer (Nano ZS, Malvern, USA) at 25 °C. The morphology and structure were obtained through Transmission electron microscope (F20, TECHNI, USA). The new synthesized nanoparticles (INPs, ARCs, and I-ARCs) were isolated from the aqueous suspension medium by Beckman OptimaTM MAX-XP Ultracentrifuge (25 000 r/min, 30 min) (Beckman, USA). The nonentrapped ICG in the supernatant was quantified to calculate the encapsulation efficiency (EE) as the formula: EE (%) = ((weight of loaded drug)/(weight of initially added drug)) ×100. Fluorescence (FL) spectra of free ICG and I-ARCs were recorded by fluorescence spectroscopy (F900, Edinburgh Instruments Ltd., UK) for 10 d. Optoacoustic signals of I-ARCs were obtained using multispectral optoacoustic tomography (MSOT) system (iThera Medical GmbH, Germany). 200 μL I-ARCs and PBS (as control) were separately injected into the channel in tissue mimic phantom. Optoacoustic signals were induced by multiple wavelength laser sources (715 nm, 730 nm, 760 nm, 800 nm and 850 nm). Then the raw optoacoustic signals were unmixed into signals of ICG, HbO_2_ and Hb. The O_2_ binding properties of free Hb, ARCs, and I-ARCs were obtained from measuring their oxygen dissociation curve^34^.

The dissolved oxygen detector (Mettler Toledo, Switzerland) measured the concentration of oxygen dissolved in solutions. Briefly, Hb, ARCs or I-ARCs (containing 4 mg mL^−1^ Hb) was prepared under pH 7.4 at 25 °C. The 1 mL samples were oxygenated to dissolved oxygen concentration of 35 mg L^−1^, while the 10 mL PBS (pH 7.4) was deoxygenated. Then the two solutions were mixed and sealed, concentration of dissolved oxygen was recorded. For thermo-stability pH stability and oxygen-carrying stability, oxygen dissociation curves of Hb, ARCs and I-ARCs were recorded under different conditions (pH 6.7, 25 °C; pH 7.4, 60 °C; 2 d, 5 d, 10 d after nanoparticles were synthesized).

### ROS and ferryl-Hb detection

The ROS production of INPs, ARCs and I-ARCs was studied using DCFH-DA as an indicator. Briefly, DCFH-DA was mixed in 96-well plate at final concentration of 10 μM with I-ARCs/INPs (containing 100 μg mL^−1^ ICG and 804 μg mL^−1^ Hb) with oxygenation or deoxygenation. After 300 s of 808 nm laser (100 mW cm^−2^) exposure, FL intensity was measured by fluorescence spectroscopy (F900, Edinburgh Instruments Ltd., UK) with emission at 522 nm and excitation at 488 nm. To quantify the ferryl-Hb, 2 mM Na_2_S was added into the solution, and the concentration of ferryl-Hb was determined by measuring the absorption at 620 nm^35^.

### *In vitro* ROS detection

MCF-7 cells were cultured in Dulbecco’s modified eagle medium (DMEM) supplemented with 10% fetal bovine serum (FBS) and 1% of penicillin and streptomycin. For *in vitro* detection of ROS generation, MCF-7 cells were incubated with DCFH-DA (10 μM) along with INPs or I-ARCs for 30 min, according to the manufacturer’s suggested protocol. After 100 mW cm^−2^ 808 nm laser irradiation for 5 min, cells were washed with PBS trice and nucleus were stained with Hoechst 33258, finally cells were then observed under a TCS SP5 laser confocal microscope (Leica, Germany). Cells were also collected to quantify the FL intensity of ROS and ICG by flow cytometry assay (QUANTA SC, Beckman, USA).

### *In vitro* quantification of ferryl-Hb

MCF-7 cells were seeded into 6-well plate (2 × 10^5 ^well^−1^), incubated with ARCs (contained 804 μg mL^−1^ Hb, ARCs were also deoxygenated for comparison), I-ARCs (contained 100 μg mL^−1^ ICG and 804 μg mL^−1^ Hb, I-ARCs were also deoxygenated for comparison) for 30 min. The cells were immediately exposed to 808 nm laser irradiation (100 mW cm^−2^) for 5 min. After treatments, cells were harvested and disrupted with sonication followed by centrifuge, and ferryl-Hb in cell extracts was quantified by measuring the absorption at 620 nm.

### *In vitro* PDT evaluation

The MCF-7 cells were seeded into 96-well plate (10^4^ well^−1^) in 150 μL of medium overnight. The medium was replaced by the medium with PBS, I-ARCs (containing 100 μg mL^−1^ ICG and 804 μg mL^−1^ Hb, I-ARCs were also deoxygenated for comparison). After 24 h incubation, the plate was placed on the 37 °C Labnet Accublock^TM^ digital dry bath. The cells were irradiated with a 100 mW cm^−2^ 808 nm laser for 5 min. After another 2 h incubation cells were washed with PBS and stained with calcein-AM for visualization of live cells and with PI for visualization of dead/late apoptotic cells. The cells were examined with biological inverted microscope (Olympus IX71, JPN).

MCF-7 cells (10^4^ well^−1^) were seeded into 96-well plate (10^4^ well^−1^), and 150 μL of INPs and I-ARCs (INPs and I-ARCs were also deoxygenated for comparison) were added and incubated for 30 min. For NIR laser treated group, the 96-well plate was exposed to 100 mW cm^−2^ 808 nm laser for 5 min. The cells were incubated for another 24 h and the cell viability was investigated by MTS assay.

### Animals and tumour model

Female BALB/c nude mice (4–6 weeks old) were purchased from Vital River Laboratory Animal Technology Co. Ltd. (China). Animals received care in accordance with the Guidance Suggestions for the Care and Use of Laboratory Animals. All the animals received care complied with the guidelines in the Guide for the Care and Use of Laboratory Animals, and the procedures of animal experiments were approved by the Institutional Ethical Committee of Animal Experimentation of Shenzhen Institutes of Advanced Technology (Chinese Academy of Sciences). The methods were carried out strictly in accordance with governmental and international guidelines on animal experimentation. All efforts were made to minimize the usage amount of animals and the suffering during experiments according to the request of Biosafety and Animal Ethics. MCF-7 cells (1 × 10^6^) were administered by subcutaneous injection into the flank region of the mice. Tumour volume was calculated as (tumour length) × (tumour width)^2^/2.

### *In vivo* FL/PA imaging and biodistribution analysis

The nude mice were randomly divided into three groups (three per group). Before administration, I-ARCs were oxygenated. Mice were intratumourally injected with 150 μL of free ICG (400 μg mL^−1^) or I-ARCs (containing 400 μg mL^−1^ ICG and 3200 μg mL^−1^ Hb). Images and FL semiquantitative analysis of ICG were taken at 0, 6, 12, and 24 h after injection using the ex/*in vivo* imaging system (CRi maestro, USA) with a 704 nm excitation wavelength and a 735 nm filter to collect the FL signals of ICG. The mice were sacrificed after injection at 24 h and the organs including heart, liver, spleen, lung, kidneys and tumour were collected for imaging and semiquantitative biodistribution analysis. For *in vivo* PA imaging, 150 μL PBS or I-ARCs (containing 400 μg mL^−1^ ICG and 3200 μg mL^−1^ Hb) was injected intratumourally at the flank region of the mice, and ICG, HbO_2_ and Hb signals in tumours were monitored using MSOT system at 715, 730, 760, 800, 850 and 900 nm (25 frames per wavelength). The raw signals were reconstructed and specific signals were spectrally resolved by the pseudo-inverse unmixing S59 method as implemented in the ViewMSOT software. For laser treatment group, 808 nm laser (100 mW cm^−2^) was irradiated for 30 min at 0.5 h postinjection.

### *In vivo* PDT

The mice (six per group) were intratumourally injected with 150 μL of PBS, free ICG + Hb (containing 400 μg mL^−1^ ICG and 3200 μg mL^−1 ^Hb), ARCs (containing 3200 μg mL^−1^ Hb), INPs (containing 400 μg mL^−1^ ICG), I-ARCs (containing 400 μg mL^−1^ ICG and 3200 μg mL^−1^ Hb), all of which were oxygenated. For laser treatment groups, the tumours of mice were irradiated by the 808 nm laser under low power (100 mW cm^−2^) for 30 min, and the temperature of tumour region was monitored, in order to avoid exceeding the thermal damage threshold (43 °C) of cancer cell^27^. Tumour volume of the mice was recorded, and the photos of mice were taken. To further detect the PDT effect *in vivo*, excised tumours at 48 h after treatment were stained with hematoxylin and eosin. On 30 d, livers and kidneys where I-ARCs had a high concentration were stained with hematoxylin and eosin for biosafety evaluation.

### Statistical analysis

Data are reported as mean ± SD. The differences among groups were determined using one-way ANOVA analysis and Student’s *t*-test; (^*^) *P* < 0.05, (^**^) *P* < 0.01.

## Additional Information

**How to cite this article**: Luo, Z. *et al.* Self-Monitoring Artificial Red Cells with Sufficient Oxygen Supply for Enhanced Photodynamic Therapy. *Sci. Rep.* **6**, 23393; doi: 10.1038/srep23393 (2016).

## Supplementary Material

Supplementary Information
