# Supplementary material for: Self-Monitoring Artificial Red Cells with Sufficient Oxygen Supply for Enhanced Photodynamic Therapy
Source: Sci Rep. 2016 Mar 18;6:23393. doi: 10.1038/srep23393 (PMC4796897; doi:10.1038/srep23393)
Supplement: Supplementary Information [file srep23393-s1.doc]

**[Supplementary Information](http://www.nature.com/srep/authors/submit.html#supplementary-info)**

Self-Monitoring Artificial Red Cells with Sufficient Oxygen Supply for Enhanced Photodynamic Therapy

Zhenyu Luo^1, 4, §^, Mingbin Zheng^1, 2, §, *^, Pengfei Zhao^1, 4^, Ze Chen^1^, Fungming Siu^3^, Ping Gong^1^, Guanhui Gao^1^, Zonghai Sheng^1^, Cuifang Zheng^1^, Yifan Ma^1, *^, and Lintao Cai^1, *^

^1^Guangdong Key Laboratory of Nanomedicine, CAS Key Lab for Health Informatics, Institute of Biomedicine and Biotechnology, Shenzhen Institutes of Advanced Technology (SIAT), Chinese Academy of Sciences, Shenzhen 518055, PR China

^2^Department of Chemistry, Guangdong Medical University, Dongguan 523808, PR China

^3^Center for High Performance Computing, Institute of Advanced Computing and Digital Engineering, Shenzhen Institutes of Advanced Technology, Chinese Academy of Sciences, Shenzhen 518055, PR China

^4^University of Chinese Academy of Sciences, Beijing 100049, PR China

^§^ These authors contributed equally to this work.

^*^Correspondence and requests for materials should be addressed to L.C. ([lt.cai@siat.ac.cn](mailto:lt.cai@siat.ac.cn)), Y.M. ([yf.ma@siat.ac.cn](mailto:yf.ma@siat.ac.cn)), and M.Z. ([mb.zheng@siat.ac.cn](mailto:mb.zheng@siat.ac.cn)).

**Supplementary figures**

**
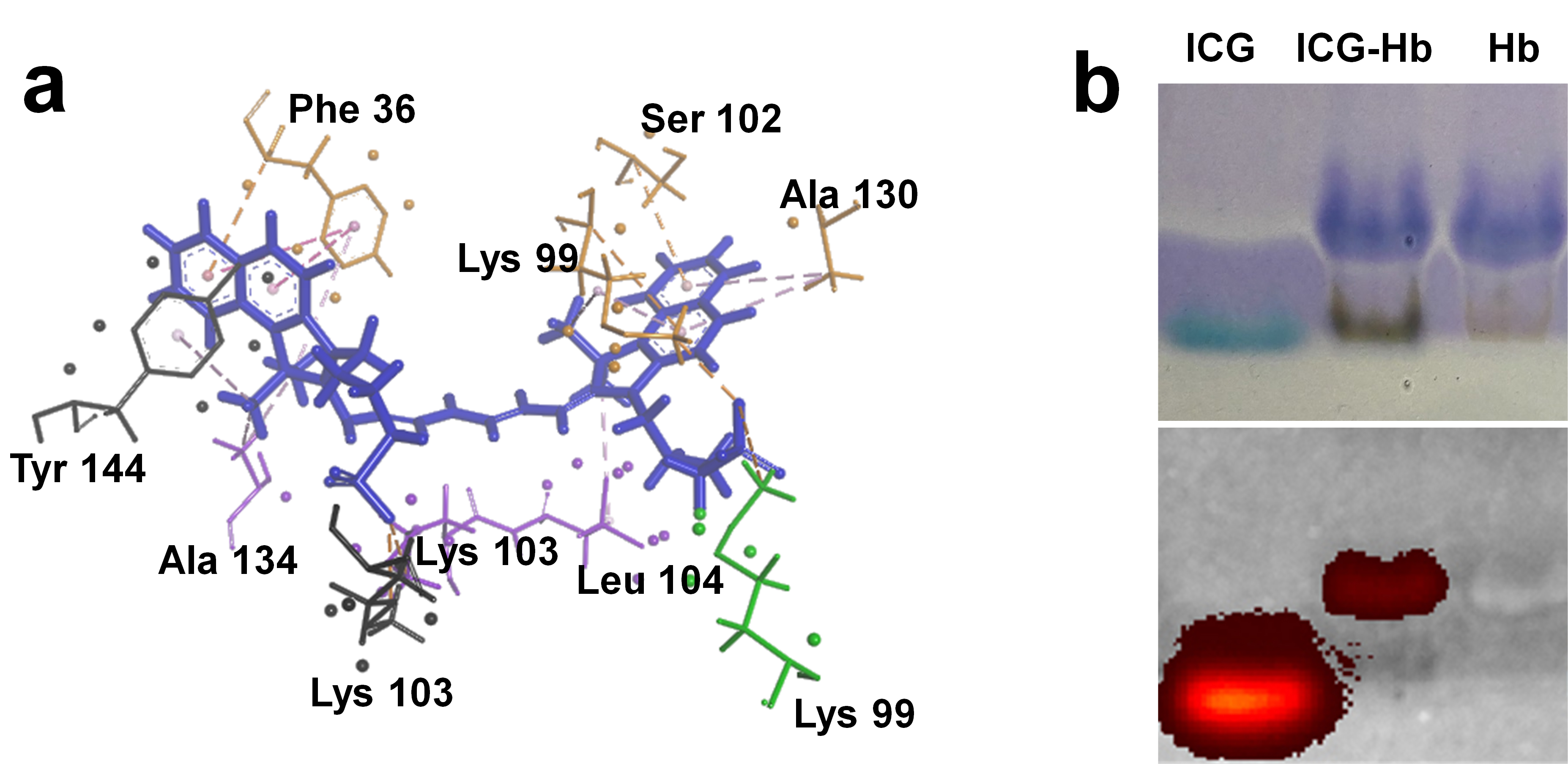
**

**Figure S1.** Binding analysis of ICG and Hb. a) The 10 contact amino acid residues representing the first interactions shell of ICG with the receptor shown by sticks. Amino acid residues of subunits A, B, C and D were colored orange, purple, green, and grey, respectively. b) Native-PAGE electrophoresis and FL imaging of ICG + Hb. The top is a brightfield photo of samples, in which Hb was stained with Coomassie Blue. The bottom is a merged picture of brightfield and FL image. The localization of ICG FL in Hb band evidenced the formation of Hb/ICG complexes.


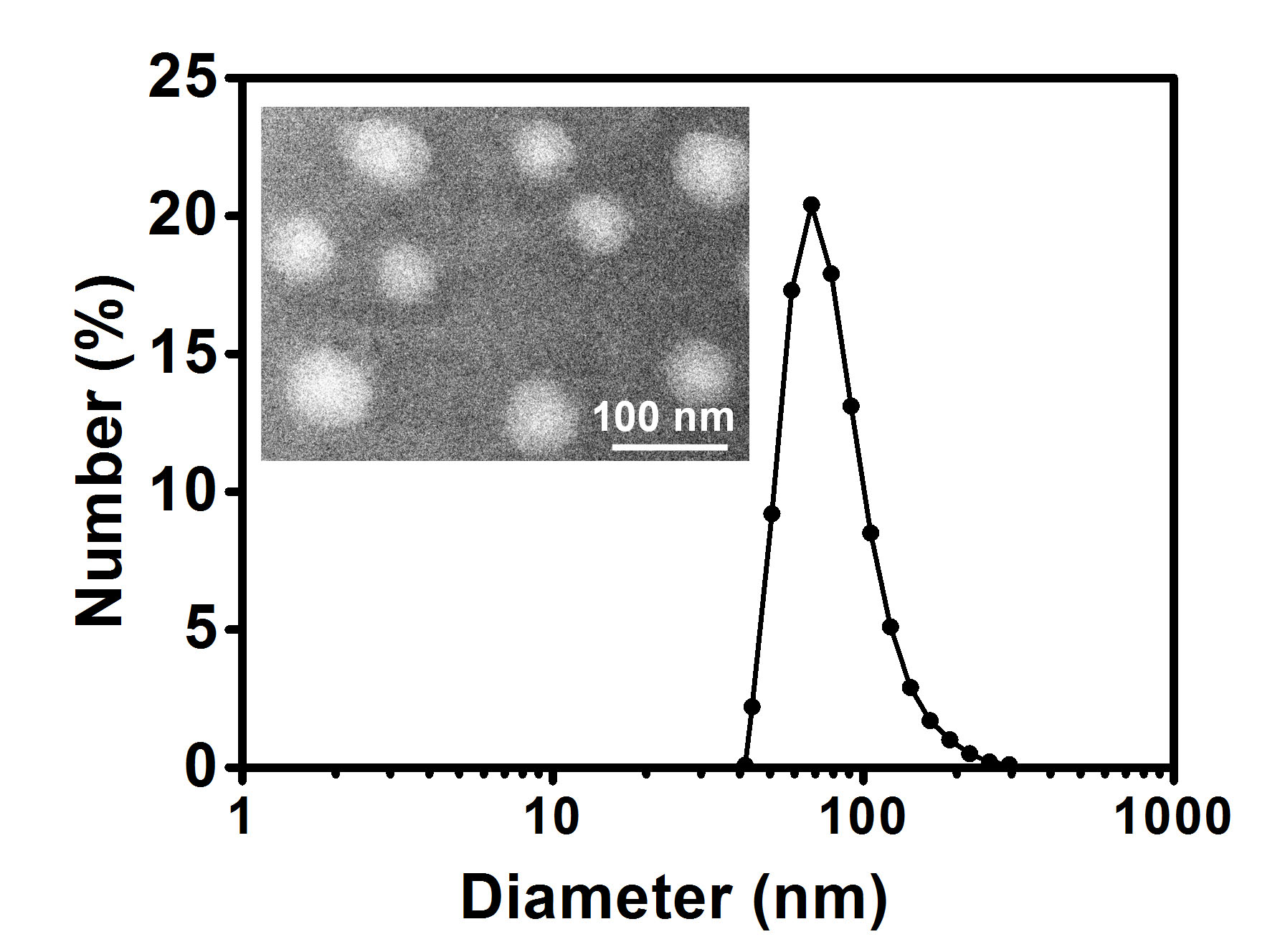


**Figure S2.** Size distribution of I-ARCs. Inset image was a TEM image of I-ARCs.


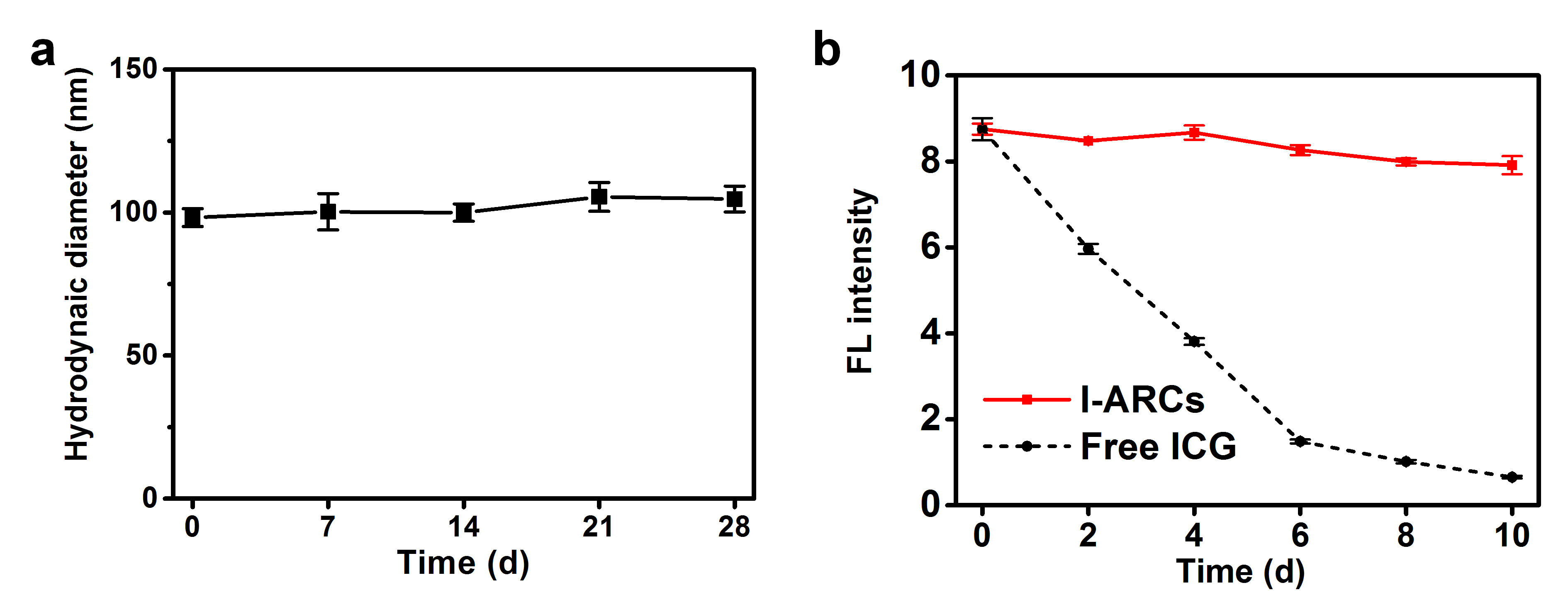


**Figure S3.** Stability of I-ARCs. a) Size stability test of I-ARCs, revealing that I-ARCs had a size stability in 28 d. b) ICG fluorescent stability of I-ARCs and free ICG. I-ARCs had better FL stability than free ICG.


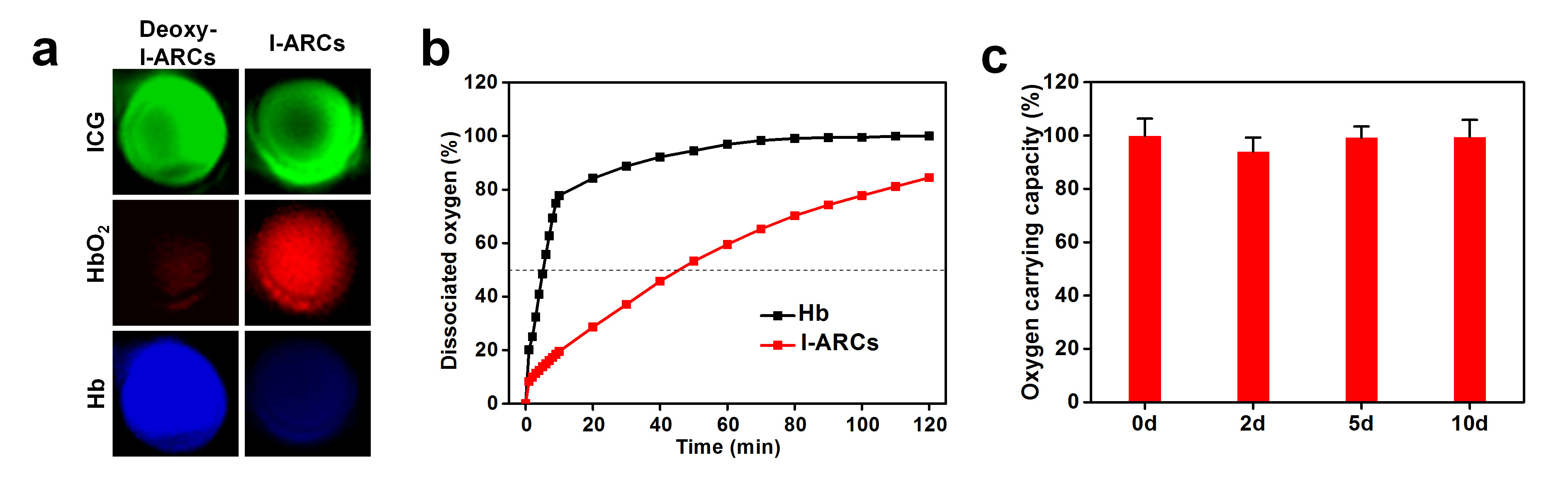


**Figure S4.** Oxygen loading capacity test of I-ARCs. a) Photoacoustic measurement of ICG (green), HbO_2_ (red) and Hb (blue) in I-ARCs. b) Oxygen dissociation curves of oxygenated I-ARCs and free Hb. The semi-dissociation time of oxygen from Hb in I-ARCs (46.0 min) is nearly 9 times of that in a free state (5.2 min). c) Evaluation of oxygen carrying capacity of I-ARCs on 0d, 2d, 5d and 10 d after I-ARCs were synthesized. Hb in I-ARCs kept its affinity to oxygen, and retained its oxygen loading function at least 10 d.

**
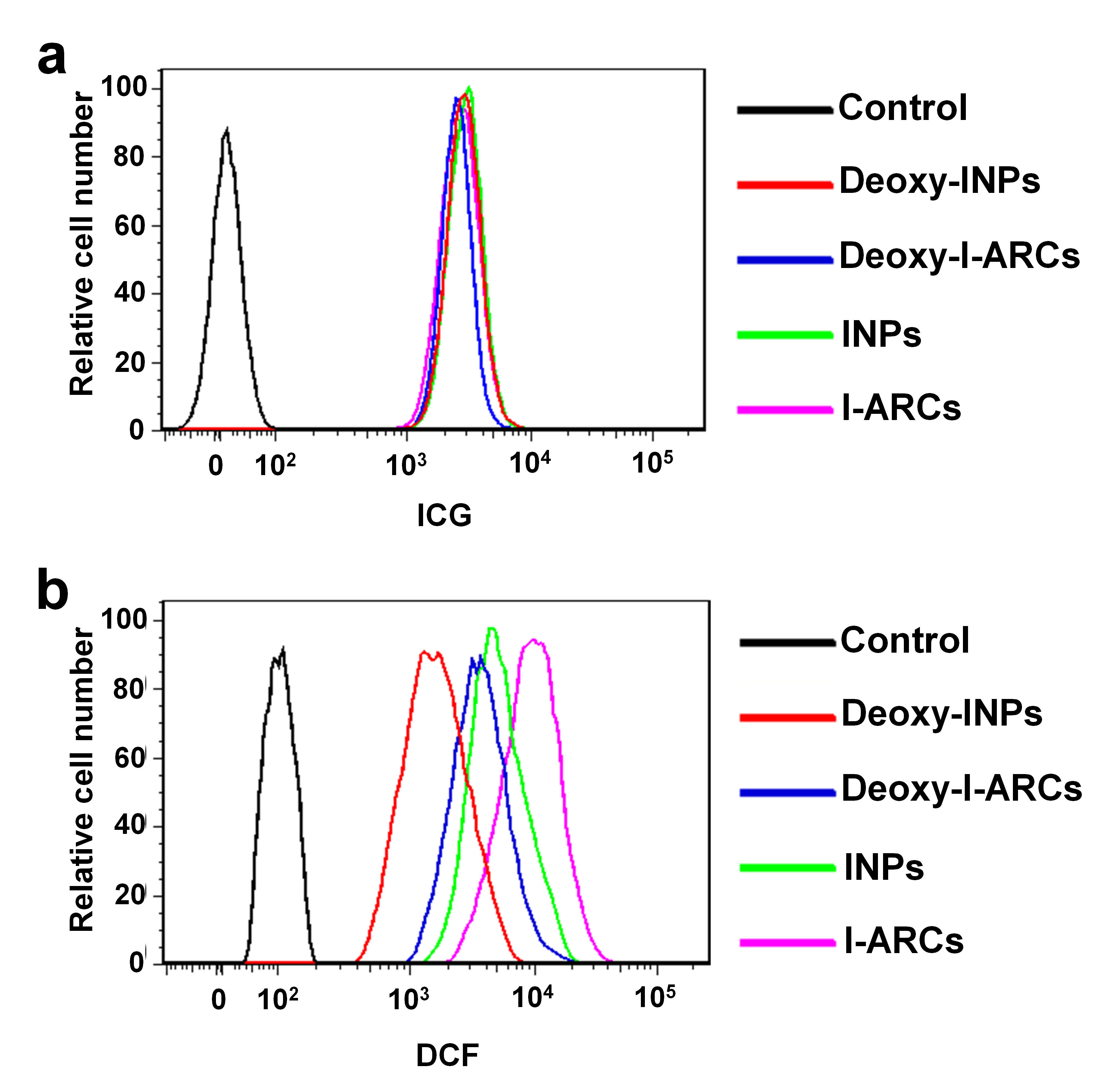
**

**Figure S5.** Cellular ROS assay after boosted PDT. a) Quantification of cellular ICG content from groups as indicated in MCF-7 cells measured by flow cytometry. b) Quantification of ROS from MCF-7 cells incubated with INPs/I-ARCs, followed by NIR laser irradiation measured by flow cytometry detecting ROS FL probe DCF. Nanoparticles were oxygenated before incubation, and for comparison, INPs and I-ARCs were also deoxygenated.

**
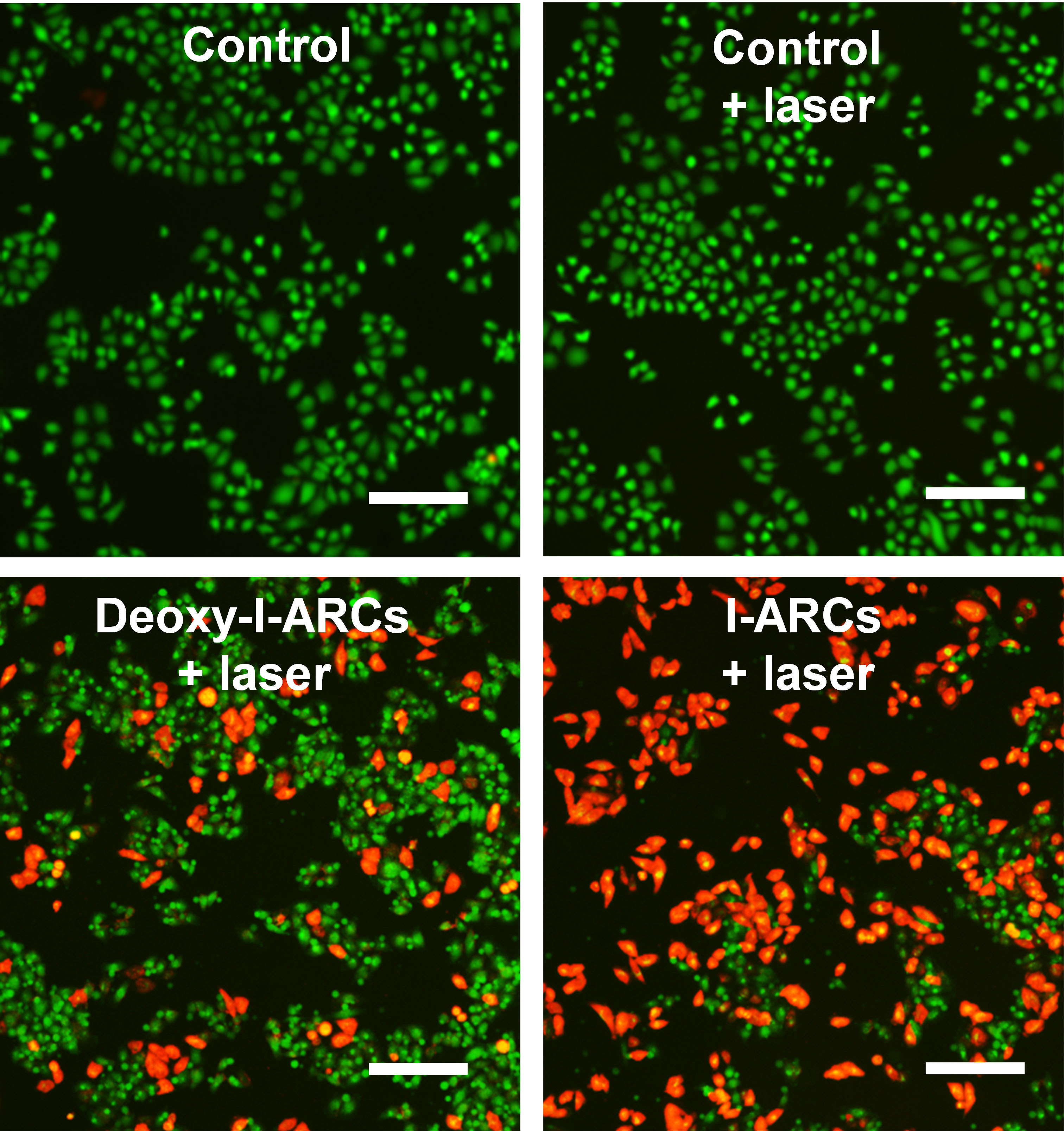
**

**Figure S6.** Necrosis assay of MCF-7 cells stained using calcein-AM and propidium iodide, indicating live cells (green) and dead cells (red), scale bars, 100μm.

**
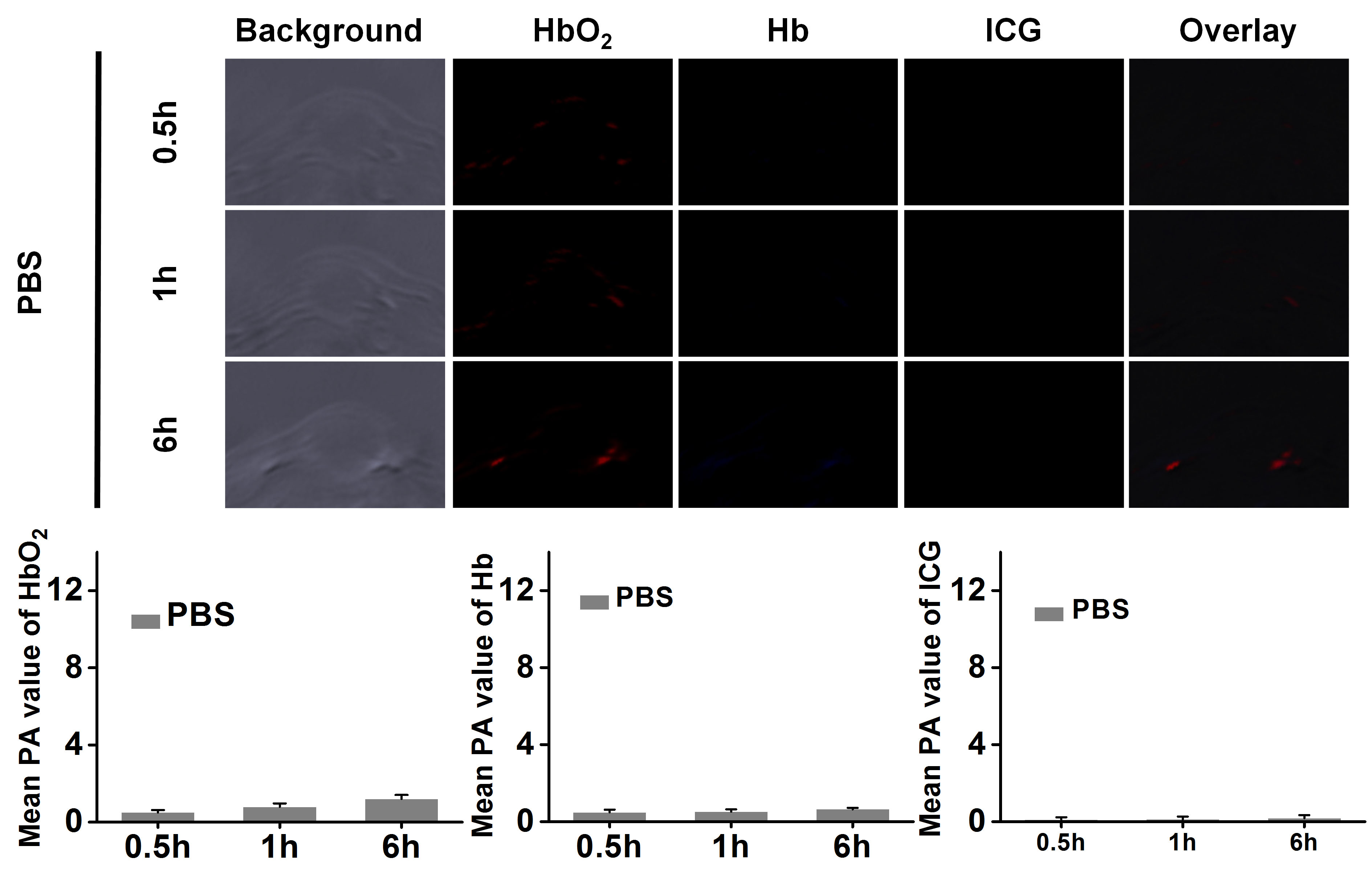
**

**Figure S7.** *In vivo* real-time PA imaging in PBS injected tumor by monitoring HbO_2_, Hb and ICG using MSOT system. *P < 0.05, **P < 0.01.

**
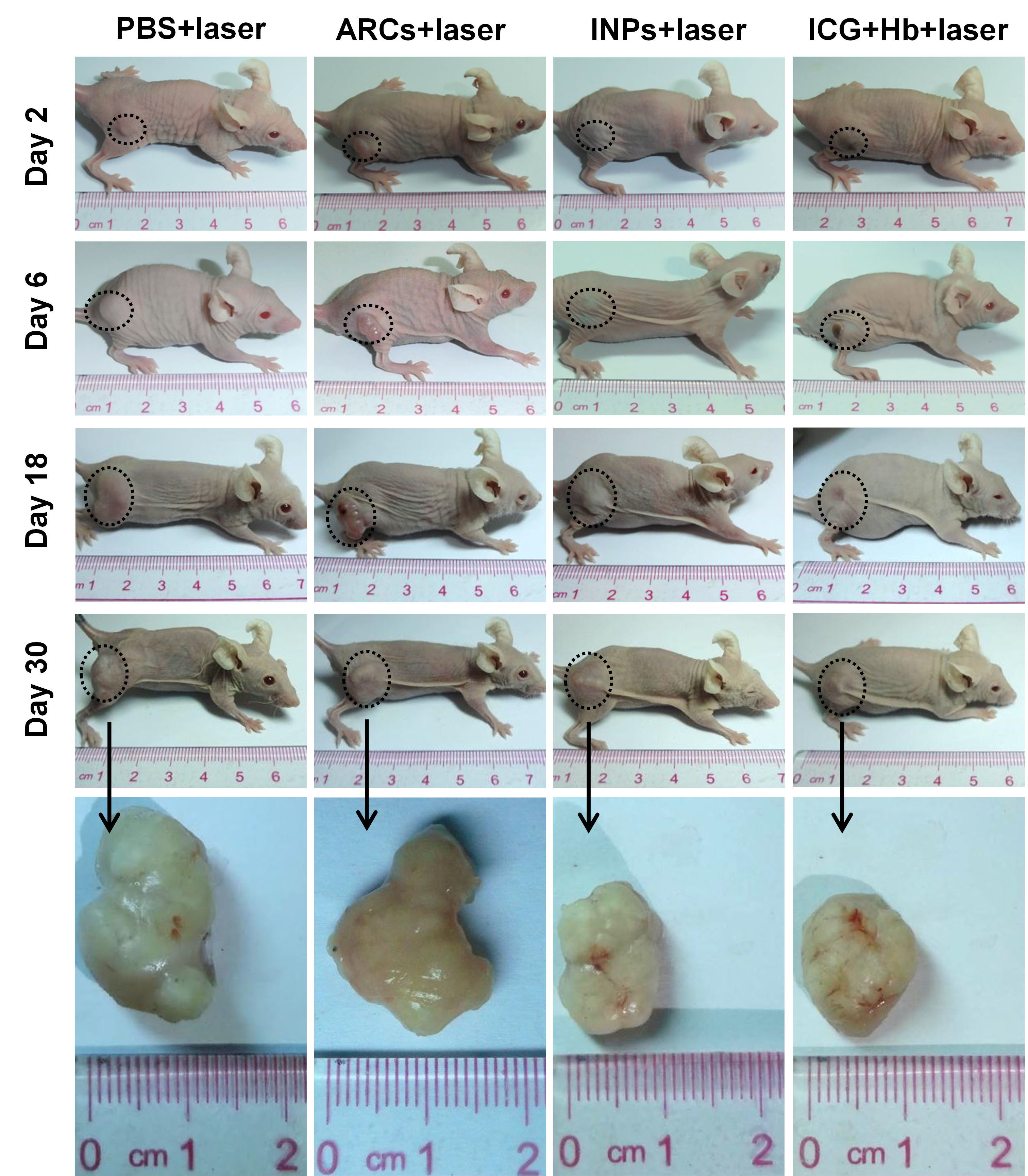
**

**Figure S8.** Representative photos of mice bearing MCF-7 tumors and excised tumors on 30 d after treatments as indicated. The tumor regions were marked with dashed circles.


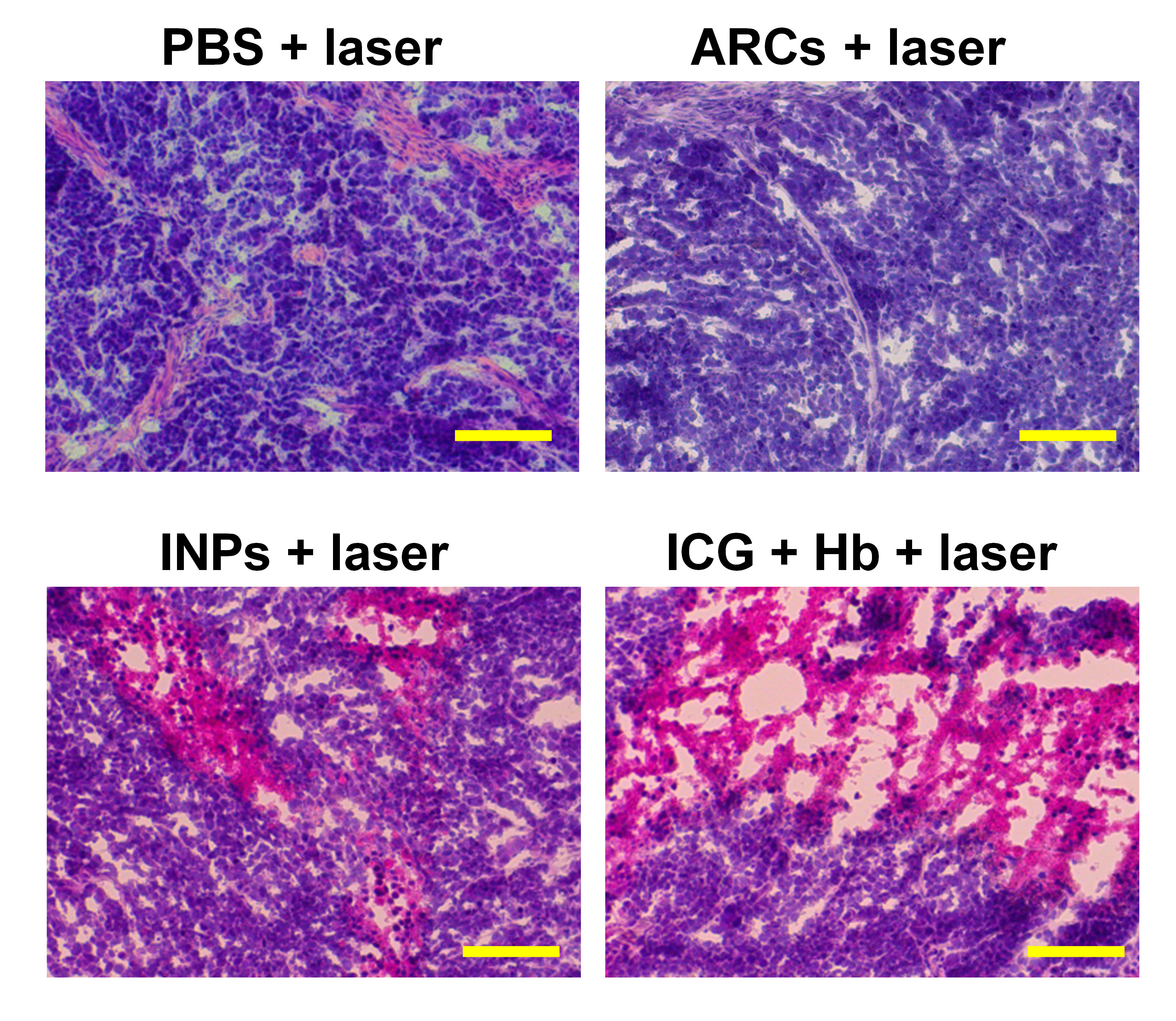


**Figure S9.** H & E stained histological sections of tumor on 2d after treated with PBS + laser, ARCs + laser, INPs +laser, ICG + Hb + laser (scale bar, 100 μm).

**
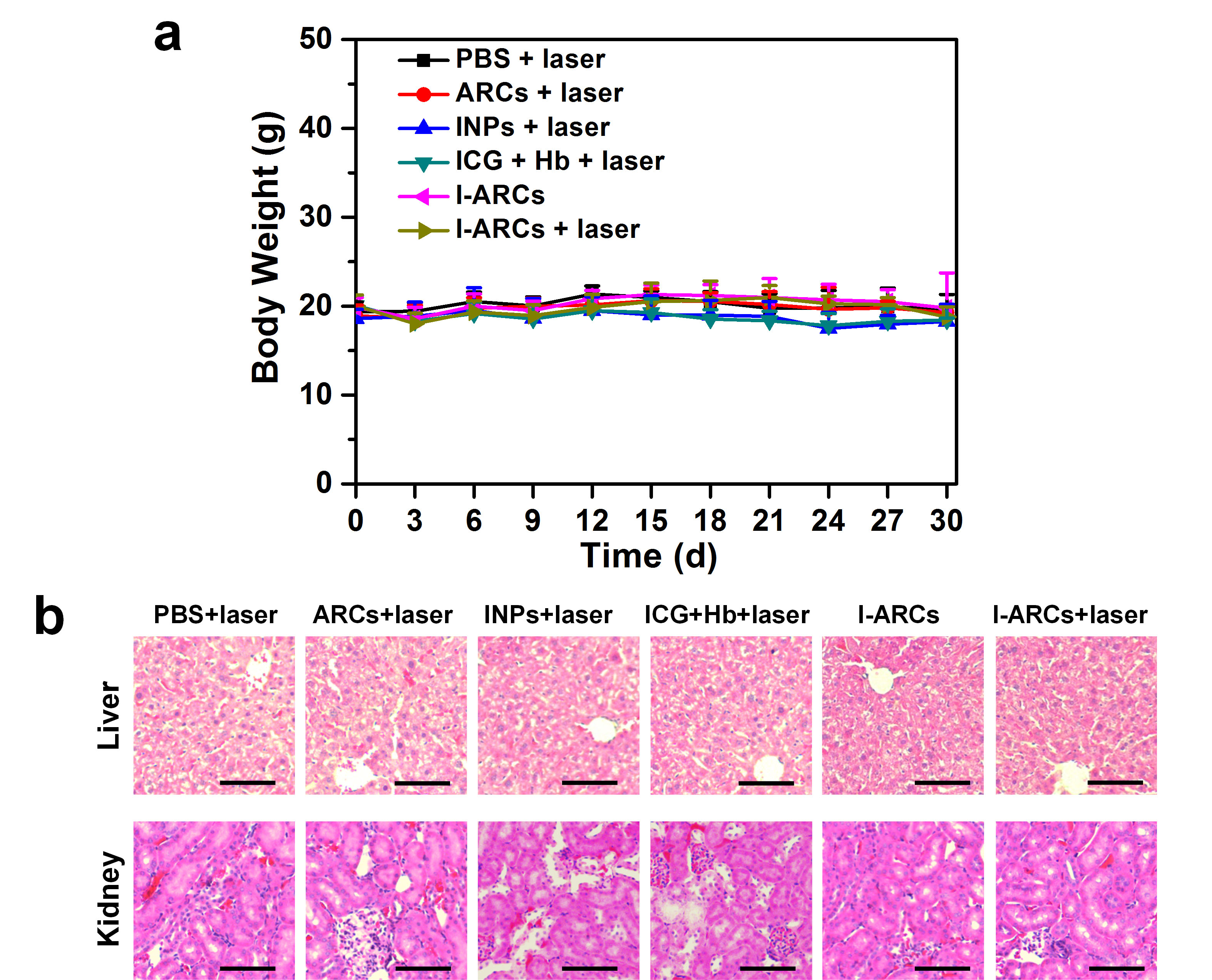
**

**Figure S10.** Biosafety evaluation. a) Body weight record of mice after treatments (n=5). b) The potential toxicity in the kidneys and liver which had significant accumulation of nanoparticles was investigated by H & E staining 30d after treatments. The H&E stained organ sections with no apparent histological changes confirmed the great biocompatibility and biosafety of I-ARCs (Scale bar, 50 μm).
